# Magnitude and Risk of Dying among Low Birth Weight Neonates in Rural Ethiopia:* A Community-Based Cross-Sectional Study*

**DOI:** 10.1155/2019/9034952

**Published:** 2019-05-16

**Authors:** Akine Eshete, Abebe Alemu, Taddes Alemu Zerfu

**Affiliations:** ^1^College of Health Sciences and Medicine, Department of Public Health, Debre Berhan University, Ethiopia; ^2^College of Health Sciences and Medicine, Department of Midwifery, Dilla University, Dilla, Ethiopia; ^3^College of Health Sciences and Medicine, Department of Public Health, Dilla University, Dilla, Ethiopia

## Abstract

**Background:**

Even if remarkable progress has been made in reducing preventable child deaths worldwide, neonatal mortality reduction has remained unsatisfactory. Low birth weight (LBW) is the major risk factor for child deaths during the neonatal period, yet only 5% of babies are weighed at birth in Ethiopia. The aim of the present study was to determine the magnitude and risk of dying among low birth weight neonates in rural Gedeo, Southern Ethiopia.

**Methods:**

Community-based mixed-method approach design was employed between September and October 2016 to identify and enroll study participants in rural Gedeo, Southern Ethiopia. Records of 17,503 live birth babies, of whom 2,065 (11.8%) had LBW, born in the last 12 months were screened to identify 885 (42.8%) biological mother–LBW neonate pairs from eight health centers. The study subjects were randomly selected using a multistage stratified cluster sampling technique. Cox proportional hazards regression model was used to predict maternal and neonatal risk factors associated with the risk of neonatal death.

**Results:**

The overall neonatal mortality rate (NMR) among LBW infants was 110 per 1000 live births (95% confidence interval: 75 –228). Close to half, 374 (42.3%), of the LBW neonates died during the first week of life. The estimated hazard ratios of mortality were higher among neonates whose mothers did not attend antenatal care (ANC) (HR=1.58, 95 % CI: 1.02-2.43), gave birth by assisted or cesarean delivery (HR=1.81 and 3.72; 95% CI: 1.10 - 3.02 and 2.11-6.55), and experienced some form of illness during pregnancy (HH=3.34, 95 % CI: 2.11-5.29), respectively. Similarly, neonates born with very low (<2000gm) birth weight and born prematurely (before 37 weeks of gestation) carried a higher (HR= 1.90 and 1.47; 95 % CI: 1.22 - 2.96 and 1.07-2.28) risk of death. On the other hand, maternal formal education was found to be the single protective factor (HR= 0.65,95 % CI: 0.43-0.99).

**Conclusion:**

Nearly one in every ten (11%) of neonates die before celebrating their firth month of life, mainly during the first week in rural Ethiopia. The risk of dying from LBW during the neonatal period is almost fourfold of the current estimated national NMR. Maternal obstetric characteristics and fetal maturity were predictors of mortality.

## 1. Introduction 

Though remarkable progress has been made in averting preventable child deaths, the neonatal mortality rate remains too high [1–3]. Promisingly, the leading causes for the vast majority of under-five deaths are infectious diseases and neonatal complications that could be readily preventable or treatable with proven, cost-effective, and quality delivered interventions [3–5]. Several studies from low- and middle-income countries [6–9] reported that neonatal death was associated with factors that could be easily implemented through the provision of a continuum of care from pregnancy to delivery and to the immediate postnatal period.

Low birth weight (LBW) is one of the key drivers and indirect causes of neonatal death [10]. It contributes to 60% to 80% of all neonatal deaths, annually [11]. A baby born with LBW is at a higher risk of morbidity [12], inhibited growth [13], poor cognitive development [11, 14], remaining underweight and stunted in early childhood [15], growing into shorter adults, and developing chronic diseases at later ages of life [16]. Low birth weight girls tend to grow into women with short stature and an underdeveloped pelvis, leading to obstetric complications during childhood [17]. According to recent estimates, each year, more than 20 million infants across the globe representing 15 to 20 percent of all life born babies have low birth weight (LBW) [13].

However, little is known about community-level factors associated with the risk progress made to reduce LBW and the associated neonatal mortality, in spite of ample evidence relating to prevention of LBW using low cost, simple, and proven technologies/interventions [13, 18]. A recent report from the lancet showed that avoiding any barrier at the time of birth could save an estimated 1.3 million newborn deaths annually by 2020 [4].

In Ethiopia, the latest (2015/16) estimate of neonatal mortality rate is 28 per 1000 live births and accounts for about half (47.5%) of the overall child mortality rate [3, 19]. The risk of neonatal death in the country varies in terms of sociodemographic and economic factors such as region, place of residence, geographic location, maternal educational status, and family wealth index [20, 21]. For example, in some regions of the country like Benishangul, Afar, Gambella, Somali, and Southern Nations, Nationalities, and People's Region, the NMR is incomparably high. Furthermore, children from rural, poor/poorest, uneducated, or less educated family (mother) carry a disproportionally higher risk of neonatal mortality [21–23].

The critical challenge of programs and interventions in Ethiopia remains to be a paucity of comprehensive community-based evidence as many of the neonatal deaths happen at home (not more than 5% of babies are weighted at birth) and left undetected [22]. Moreover, few of the available studies are often facility-based or investigated neonatal mortality as a whole, not inferring LBW babies in particular. To the best of our knowledge, this is the first community-based study investigating the burden and predictors of mortality among LBW neonates.

Therefore, the present study aimed to generate and analyze community-based data on the magnitude and associated maternal and neonatal risk factors of neonatal death among LBW neonates in rural communities of Gedeo zone, Southern Ethiopia. The study findings will be used as inputs for policy makers and program implementers to design and implement context-based intervention to tackle the problem.

## 2. Methods and Materials

We employed a community-based cross-sectional quantitative study design to collect data on magnitude and LBW related death risks between September and October 2016. The study was conducted in three randomly selected districts of the Gedeo zone in the Southern Nations, Nationalities, and People's (SNNP) Regional State of Ethiopia. The zone has six districts and two administrative towns (namely, Dilla and Yirgachefe towns) and is known for leading coffee producing areas in the country. We selected three districts and six clusters or kebeles (smallest administrative units) for the study.

### 2.1. Ethics Statement

The study was approved by the Health Research and Ethics Review Committee of the College of Health Sciences of Dilla University. Written informed consent was obtained from the participants. The information obtained was kept anonymous, thereby ensuring confidentiality.

### 2.2. Recruitment and Interviewing of Study Participants

The study population comprises pairs of mothers and LBW infants born alive 12 months prior to the data collection period. The infants were included only when they were living with their biological mothers, having birth certificates, apparently healthy, and born with a birth weight of <2500gm.

A multistage cluster sampling technique was employed to select and enroll mothers in the study. The stratification was into urban and rural strata, followed by the use of simple random sampling to identify three districts and study clusters (kebeles). A total of six clusters (two from each district) were randomly selected and included to the study. Form the selected clusters, all neonates were included in the study; this yielded a total sample of 885 mother–LBW neonate pairs.

Data were collected with face to face interview questionnaires. The questionnaires were designed and modified in comparison with previous studies [6, 8]. Data were collected by twelve health extension workers and four supervisors after three days of intensive training on interviewing techniques and measurements. Completed questionnaires were reviewed daily by the supervisors and principal investigators.

The dependent variable, neonatal death, was determined by recording the death of neonates within the first 28 days in the surveyed households and predicting variables from maternal and neonatal risk factors for neonatal death. Neonatal death is defined as death before 28 completed days. The date of death was recorded and infants who lived beyond 28 days were also censored at the time of data collection in completing of survival analysis.

### 2.3. Data Management and Analysis

Data were entered using Epi INFO vision 3.5.1 and exported to SPSS V-20 for analysis. Cox proportional hazards model was used to investigate maternal and neonatal risk factors for neonatal death. Explanatory variables with a p < 0.20 in univariate Cox-regression analysis were further included in the multiple-Cox-regression analysis. Finally, the multiple-Cox-regression analysis was used to estimate hazard ratios (HR); corresponding with its 95 % confidence intervals (CI).

## 3. Results 

### 3.1. Characteristics of Mothers and Neonates

Overall, 885 mother-neonate pairs were included in the study. Nearly half, 380 (43%), of mothers were 24-29 years old with a mean (± SD) and a median age of 28.5 (± 4.8) and 28.0 years, respectively. More than half, 518 (58.5%), of mothers did not attend formal education and 75.5% were housewives. Even though most, 688 (77.7%), of mothers reported to have at least one antenatal care (ANC) visit during their last pregnancy, less than one-fifth, 126(18.3%), had four or more ANC visits. The proportion of twin births was 4.4% (Table 1).

### 3.2. Magnitude and Timing of Death among LBW Neonates

From the total 885 LBW neonates born during the study period, over one in every ten 97 (10.9%), 95% confidence interval (CI), 8.8-13.1) died before completing 28 days of life. A higher number of deaths were recorded during the first 41(4.6%), second 25 (2.8%), and third 24 (2.7%) weeks of neonatal life (Table 2).

### 3.3. Neonatal Death and Maternal Characteristics

A higher proportion of neonatal death was observed among mothers aged >30yrs, 46 (47.7%); married, 85 (87.6%); being unemployed/housewives, 61 (62.9%); attending fewer (<4) ANC visits, 47 (83.9%); having spontaneous vaginal delivery, 44 (45.4%); and experiencing some form of illness during pregnancy, 58 (59.8%) (Table 3). Similarly, neonates who had very low (<2000gm) birth weight and twins also had a higher risk of death (Table 4).

### 3.4. Risk Factors for Neonatal Death among Low Birth Weight Babies

A proportional Cox-regression analysis was employed to examine maternal and newborn factors affecting neonatal death, and results are presented in Tables 5 and 6. In binary Cox-regression analysis, neonates whose mothers did not have ANC follow-up visit (HR=2.67, 95 % CI: 1.78 -3.98), were grand multiparous (have more than six birth) (HR=2.19, 95 %; CI: 1.28, 3.74), and had maternal illness during perinatal period (HR=5.28, 95 %; CI: 3.52, 7.93) carried a high risk of neonatal death. In addition, mothers who had either instrumental or cesarean delivery carried a three- to sevenfold (HR=3.36 and 7.44; 95 % CI: 2.12, 5.35 and 4.49-12.32) higher risk of neonatal death compared to mothers who had a spontaneous vaginal delivery (SVD) (Table 5). Neonates born before 37 weeks of gestation, in multiple births, and with birth weight < 2000gm had a 1.81 (HR= 1.81, 95 % CI: 1.19-2.75), 3.07 (HR= 3.07,95 % CI: 1.66-5.75), and 3.08 (HR= 3.08,95 % CI: 2.07-4.60) higher risk of death.

During the multivariate-adjusted hazard analysis, maternal education (HR= 0.65, 95 % CI: 0.43-0.99), instrumental (HR=1.81, 95% CI: 1.10-3.02) or cesarean delivery (HR=3.72, 95 % CI: 2.11- 6.55), lack of ANC follow-up visits (HR=1.58, 95 % CI: 1.02-2.43), and experiencing some form of illness during pregnancy (HR=3.34, 95% CI: 2.11-5.29) remained as independent maternal risk factors predicting higher risk of death among LBW neonates (Table 5).

In the same way, prematurity (HR= 1.47, 95 % CI: 1.07-2.28) and very low birth weight at birth (HR= 1.90, 95 % CI: 1.22-2.96) were neonatal conditions with independent predictors of higher risk of mortality of LBW infants.

## 4. Discussion

In this paper, we described the results of a community-based cross-sectional study that aimed to determine the magnitude and predictors of death among LBW neonates in rural districts of Gedeo, Southern Ethiopia. We found that nearly one in every ten (11%) (95 % CI: 17.5 - 22.8) LBW infants were dying before celebrating their first month of life (neonatal period). We observed that the risk of death was much higher during the first week of life. Both maternal and neonatal factors were significantly associated with the risk of death of an LBW neonate. Maternal obstetric characteristics like too few or no ANC follow-up visits, assisted/caesarian delivery, and experience of some form of illness during pregnancy and neonatal birth conditions like prematurity and having very low birth weight at birth were independent predictors of mortality among LBW neonates.

The magnitude of neonatal death (110 per 1000 LB) observed among LBW neonates in the current study is almost a fourfold of the overall estimated national neonatal mortality rate (28 per 1000 LB) for Ethiopia [3] and exceeds the rate for similar middle-income city, Cuiaba, of Brazil by 63.6% [24]. On the other hand, it is quite incomparable and too much higher (almost 5-11-fold) than the risk of death among twins and LBW infants of a population-based study of developed countries, England and Wales. Rather it is comparable to the death rate of very low birth weight (VLBW) neonates of the same country [25, 26]. This shows that the observed mortality rate among LBW neonates in rural Ethiopia is quite very high and may be one of the highest in the world [3, 20–23].

The main reason for such unacceptably high mortality among LBW neonates could be related to poor healthcare seeking behavior of mothers (caretakers) and/or families for neonatal and childhood health problems in such settings [27, 28]. In addition, low availability and poor quality [29, 30] of child healthcare facilities in the area could further worsen the risk of death of LBW neonates.

Though we did not assess the causes of neonatal deaths using verbal autopsy, we could see that the leading predictors of death were related to maternal sociodemographic and obstetric characteristics that could be easily improved by providing a continuum of care to women during pregnancy, labor, immediate, and early neonatal periods [20]. Evidence suggests that three million babies and women could be saved each year by investing in quality care at the time of birth and special care for sick and small newborns; specifically care for low birth weight and sick newborns could avert 30 % of neonatal deaths.

In this study, higher neonatal deaths occurred in the first week of life 42.3 (95% CI: 33.0-51.5), compared to the second 25.8 (95% CI: 17.5-35.1), third 24.7 (95% CI: 16.5-33.0), and fourth weeks 7.2 (95% CI: 2.1-13.4), which was consistent with previous findings [24–26]. The rate of death among LBW neonates was higher among mothers who had less than 4 ANC visits (83.9%), had nonspontaneous assisted or caesarian delivery (54.6%), and experienced illness during pregnancy (59.8%). Similarly, neonates born with very low birth weight (< 2000gm) carried a higher (54.6%) risk of death compared to others (Table 3).

Neonates whose mothers attended formal education had a lower risk of death, which agreed with the studies done in Ethiopia [25] and Nigeria [16]. The reason may be that educated mothers have awareness of the advantages of maternal healthcare service utilization to both mother and their babies, and that has an effect on child survival in the postnatal period. Even though birth order and birth interval had statistical significance with neonatal death in previous studies [16, 26, 28], these variables had no association with neonatal death in this study.

Similar to previous findings [24–29], the present study also revealed that the risk of neonatal death was higher among twins. However, after adjusting for other variables, the risk of death did not vary significantly by type of birth. This is mainly due to the fact that small size at birth and prematurity are major factors that increase the risk of neonatal death as premature, twining, and small size births are more likely to develop complications [16].

Inadequate or lack of ANC visits and occurrence of maternal illness were other maternal predictors for early neonatal deaths among LBW neonates in the study area. Several previous studies [26–29] have also reported lack of adequate and quality ANC visits that results in the inadequate monitoring of pregnancy, maternal, and neonatal complication during and after delivery, which was associated with increased risk of neonatal death. This suggests that special consideration needs to be given to maternal obstetric care during pregnancy.

LBW neonates born through assisted (instrumental or cesarean) delivery were also found to have two and four times added risk of dying compared to those born spontaneously. This could be related to elevated risk of infection and complications among nonspontaneous assisted deliveries as most are conducted after long-obstructed labor, usually referred from other facilities [25].

The study has several strengths that should be appreciated. Firstly, it is among the very few studies conducted to assess prevalence and risk factors of death among LBW neonates. Secondly, unlike the existing evidence often based on facility-based studies, the resent study employed community-based study design in an area where a birth registry is very uncommon. The study also made use of available local evidence to strengthen evidence generated. On the other hand, the study also has limitations that should be taken into consideration while interpreting the findings. We used our own operational definition for very low birth weight (<2000g), unlike the conventional definition of a baby born < 1500g. This was made deliberately due to the fact that we have very few cases of the conventional definition leaving data analysis difficult or with poor meaningful interpretation. In addition, our study measured birth weight from health facility records which are not validated by concerned authorities for consistency and accuracy

## 5. Conclusions

In summary, the present study reported that the magnitude of neonatal mortality among LBW neonates was 11.0% (95% CI: 17.5-22.8), which is one of the highest in the world and a fourfold of the national estimated neonatal mortality rate. The first week of life is the riskiest period for survival of LBW neonates. The risk of dying among LBW neonates is determined by both maternal sociodemographic and obstetric characteristics, and neonatal birth conditions. Maternal antenatal care attendance, mode of delivery, and experience of illnesses during pregnancy were among the maternal independent predictors of mortality among LBW neonates. Very low (<2000gm) birth weight and prematurity are neonatal independent predictors of death among LBW neonates in rural settings of Southern Ethiopia.

## Figures and Tables

**Table 1:** Selected sociodemographic and reproductive characteristics of mothers and neonates, rural Gedeo, Southern Ethiopia, 2016.

Variables	Number (%)^n^
Age of mother at last birth	
≤24 Years	177 (20.0)
24-29 Years	380 (42.9)
≥30	328 (37.1)
Ethnicity group	
Gedeo	793 (89.5)
Others	93 (10.5)
Educational status of mother	
Uneducated	518 (58.5)
Educated	367 (41.5)
Occupational status of mother	
House wife	668 (75.5)
Employed (Self, Government and Private)	217 (24.5)
Sex of Neonate	
Male	431 (48.7)
Female	454 (51.3)
Types of Birth	
Singleton	846 (95.6)
Twins	39 (4.4)
ANC Follow-Up	
Yes	688 (77.7)
No	197 (22.3)
Number of ANC Visits	
<4 Visits	562 (81.7)
4^+^ Visits	126 (18.3)

n = 885

**Table 2:** Timing of neonatal death among infants of low birth weight in rural districts of Gedeo, Southern Ethiopia, 2016.

Events	Death (n)	95 % CI
Neonatal deaths in 1^st^ week	41	42.3 (33.0-51.5)
Neonatal deaths in 2^nd^ week	25	25.8 (17.5-35.1)
Neonatal deaths in 3^rd^ week	24	24.7 (16.5-33.0)
Neonatal deaths in 4^th^ week	7	7.2 (2.1-13.4)
Early neonatal death	43	42.3 (32.1-52.6)
Late neonatal death	56	57.7 (47.4-67.9)
Total neonatal deaths	97	11.0 (8.8-13.1)

**Table 3:** Distribution of neonatal deaths described by maternal sociodemographic and obstetric characteristics, rural Gedeo, Southern Ethiopia, 2016.

Variables	Total N (%)	Death N (%)
Age		
≤24 years	177 (20.0)	14 (14.4)
24-29 years	380 (42.9)	37 (38.1)
≥30	328 (37.1)	46 (47.7)
Educational status		
No formal education	518 (58.5)	49 (50.5)
Formal education	367 (41.5)	48 (49.5)
Marital status		
Married	827 (93.4)	85 (87.6)
Other	58 (6.6)	12 (12.4)
Occupational		
House wife	668 (75.5)	61 (62.9)
Employed	217 (24.5)	36 (37.1)
ANC follow up		
Yes	688 (77.7)	56 (57.7)
No	197 (22.3)	41 (42.3)
Number of ANC visits (n=688)		
< 4 ANC visits	562 (81.7)	47 (83.9)
≥4 ANC visits	126 (18.3)	9 (16.1)
Mode of delivery		
Vaginal delivery	681 (76.9)	44 (45.4)
Assisted delivery	148 (16.7)	30 (30.9)
Cesarean section	56 (6.3)	23 (23.7)
Illness during pregnancy		
Yes	214 (24.2)	58 (59.8)
No	671 (75.8)	39 (40.2)

**Table 4:** Distribution of neonatal deaths described by neonatal birth characteristics, rural Gedeo, Southern Ethiopia, 2016.

Variables	Total N (%)	Death N (%)
Preceding birth interval (year)		
First birth	125 (14.1)	10 (10.3)
<2 years	327 (36.9)	40 (41.2)
>3 years	433 (48.9)	47 (48.5)
Birth order		
< 2nd birth	324 (36.6)	26 (26.8)
3rd-5th birth	404 (45.6)	44 (45.4)
> 6th birth	157 (17.7)	27 (27.8)
Sex of neonate		
Male	431 (48.7)	49 (50.5)
Female	451 (51.3)	48 (49.5)
Types of birth		
Singleton	846 (95.6)	86 (88.8)
Twins	39 (4.4)	11 (11.3)
Weight of neonates		
< 2000 grams	195 (22.0)	44 (45.4)
> 2000 grams	690 (78.0)	53 (54.6)
Gestation age at birth		
Preterm (< 37 weeks	201 (22.7)	33 (34.0)
Term (>37 weeks)	684 (77.3)	64 (66.0)

**Table 5:** Proportional Cox-regression analysis of maternal risk factors associated with neonatal death in rural districts of Gedeo Zone, Southern Ethiopia, 2016.

Variables	Total (n=885)	Death n=97 (n = %)	*Crude HR *		*Adjusted HR *	
			95 % CI	P -values	95 % CI	P -values
Maternal age at birth						
≤24 years	177	14 (7.9)	0.80 (0.43-1.48)	0.473	0.81 (0.41-1.63)	0.555
24-29 years	380	37 (9.7)	1(Ref)		1(Ref)	
≥30	328	46 (14.0)	1.44 (0.94-2.23)	0.094	1.05 (0.62-1.81)	0.848
Educational status						
Non educated	518	49 (9.5)	1(Ref)		*1*(Ref)	
Educated	367	48 (13.1)	0.71 (0.48-1.06)	0.091	*0.65 (0.43-0.99)*	*0.049*
ANC follow-up						
Yes	688	56 (8.1)	1(Ref)		1(Ref)	
No	197	41 (20.8)	*2.67 (1.78-3.98)*	*<0.001*	*1.58 (1.02-2.43)*	*0.040*
Mode of delivery						
Vaginal delivery	681	44 (6.5)	1(Ref)		1(Ref)	
Assisted delivery	148	30 (20.3)	*3.36 (2.12-5.35)*	*<0.001*	*1.82 (1.10-3.02)*	*0.020*
Cesarean section	56	23 (41.1)	*7.44 (4.49-12.32)*	*<0.001*	*3.72 (2.11-6.55)*	*0.001*
Maternal illness during perinatal						
Yes	214	58 (27.1)	*5.28 (3.52-7.93)*	*<0.001*	*3.34 (2.11-5.29)*	*0.001*
No	671	39 (5.8)	1(Ref)		1(Ref)	

**Table 6:** Proportional Cox-regression analysis of newborn related risk factors associated with death, rural Gedeo Zone, Southern Ethiopia, 2016.

Variables	Total (n =885)	Death^1^ (n /%)	*Crude HR*		*Adjusted HR*	
			95 % CI	P -values	95 % CI	P-values
Birth interval (year)						
First birth	125	10 (8.0)	0.73 (0.38-1.45)	0.367		
<2 years	327	40 (12.2)	1.12 (0.74-1.71)	0.590		
>3 years	433	47 (10.9)	1(Ref)			
Birth order						
< 2nd birth	324	26 (8.0)	0.72 (0.44-1.17)	0.187	1.28 (0.70-2.33)	0.425
3rd-5th birth	404	44 (10.9)	1(Ref)		1(Ref)	
> 6th birth	157	27 (17.2)	1.58 (0.98-2.55)	0.063	1.04 (0.59-1.83)	0.900
Sex						
Male	431	49 (11.4)	1.09 (0.73-1.61)	0.700		
Female	454	48 (10.6)	1(Ref)			
Types of birth						
Singleton	846	86 (10.2)	1(Ref)		1(Ref)	
Twins	39	11 (28.2)	*3.07 (1.64-5.75)*	*<0.001*	1.49 (0.77-2.91)	0.239
Weight of neonates						
<2000 grams	195	44 (22.6)	*3.08 (2.07-4.60)*	*<0.001*	*1.90 (1.22-2.96)*	*0.005*
< 2000 grams	690	53 (7.7)	*1*(Ref)		*1*(Ref)	
Gestation age at birth						
Preterm (< 37 weeks)	201	33 (16.4)	*1.81 (1.19-2.75)*	*0.006*	*1.47 (1.07-2.28)*	*0.043*
Term (≥37 weeks)	684	64 (9.4)	*1*(Ref)		*1*(Ref)	
Feeding of babies within 28 days						
Only breast milk	509	50 (11.0)	1(Ref)			
With additional food	376	47 (12.5)	1.27 (0.85-1.89)	0.236		

^1^n = 97.

## Data Availability

The authors confirm that all relevant data were included in the manuscript and the raw dataset can be obtained by email request to akine.eshete@yahoo.com.
